# A Scalable Bayesian Sampling Method Based on Stochastic Gradient Descent Isotropization

**DOI:** 10.3390/e23111426

**Published:** 2021-10-28

**Authors:** Giulio Franzese, Dimitrios Milios, Maurizio Filippone, Pietro Michiardi

**Affiliations:** Data Science Department, Eurecom, 06410 Biot, France; dimitrios.milios@eurecom.fr (D.M.); maurizio.filippone@eurecom.fr (M.F.); pietro.michiardi@eurecom.fr (P.M.)

**Keywords:** Bayesian sampling, stochastic gradients, Monte Carlo integration

## Abstract

Stochastic gradient sg-based algorithms for Markov chain Monte Carlo sampling (sgmcmc) tackle large-scale Bayesian modeling problems by operating on mini-batches and injecting noise on sgsteps. The sampling properties of these algorithms are determined by user choices, such as the covariance of the injected noise and the learning rate, and by problem-specific factors, such as assumptions on the loss landscape and the covariance of sg noise. However, current sgmcmc algorithms applied to popular complex models such as Deep Nets cannot simultaneously satisfy the assumptions on loss landscapes and on the behavior of the covariance of the sg noise, while operating with the practical requirement of non-vanishing learning rates. In this work we propose a novel practical method, which makes the sg noise isotropic, using a fixed learning rate that we determine analytically. Extensive experimental validations indicate that our proposal is competitive with the state of the art on sgmcmc.

## 1. Introduction

Stochastic gradient (sg) methods have been extensively studied as a means for mcmc-based Bayesian posterior sampling algorithms to scale to large data regimes. Variants of sg-mcmc algorithms have been studied through the lens of first [1,2,3] or second-order [4,5] Langevin Dynamics, which are mathematically convenient continuous-time processes that correspond to discrete-time gradient methods with and without momentum, respectively. The common traits underlying many methods from the literature can be summarized as follows: they address large data requirements using sg and mini-batching, they inject Gaussian noise throughout the algorithm execution, and they avoid the expensive Metropolis-Hasting accept/reject tests that use the whole data [1,2,4].

Despite mathematical elegance and some promising results restricted to simple models, current approaches fall short in dealing with the complexity of the loss landscape typical of popular modern machine learning models, e.g., neural networks [6,7], for which stochastic optimization poses some serious challenges [8,9].

In general, sg-mcmc algorithms inject random noise to sg descent algorithms: the covariance of such noise and the learning rate, or step-size in the stochastic differential equation simulation community, are tightly related to the assumptions on the loss landscape, which together with the sg noise, determine the sampling properties of these methods [5]. However, current sg-mcmc algorithms applied to popular complex models such as Deep Nets, cannot simultaneously satisfy the assumptions on posterior distribution geometry and on the behavior of the covariance of the sg noise, while operating with the practical requirement of non-vanishing learning rates. In this paper, in accordance with most of the Neural Network related literature, we refer to the posterior distribution geometry as loss landscape. Some recent work [10], instead, argue for fixed step sizes, but settle for variational approximations of quadratic losses. Although we are not the first to highlight these issues, including the lack of a unified notation [5], we believe that studying the role of noise in sg-mcmc algorithms has not received enough attention, and a deeper understanding is truly desirable, as it can clarify how various methods compare. Most importantly, this endeavor can suggest novel and more practical algorithms relying on fewer parameters and less restrictive assumptions.

In this work we chose a mathematical notation that emphasizes the role of noise covariances and learning rate on the behavior of sg-mcmc algorithms (Section 2). As a result, the equivalence between learning rate annealing and extremely large injected noise covariance becomes apparent, and this allows us to propose a novel practical sg-mcmc algorithm (Section 3). We derive our proposal, by first analyzing the case where we inject the smallest complementary noise such that its combined effects with the sg noise result in an isotropic noise. Thanks to this isotropic property of the noise, it is possible to deal with intricate loss surfaces typical of deep models, and produce samples from the true posterior without learning rate annealing. This, however, comes at the expense of cubic complexity matrix operations. We address such issues through a practical variant of our scheme, which employs well-known approximations to the sg noise covariance (see, e.g., [11]). The result is an algorithm that produces approximate posterior samples with a fixed, theoretically derived, learning rate. Please note that in generic Bayesian deep learning setting, none of the existing implementations of sg-mcmc methods converge to the true posterior without learning rate annealing. In contrast, our method automatically determines an appropriate learning rate through a simple estimation procedure. Furthermore, our approach can be readily applied to pre-trained models: after a “warmup” phase to compute sg noise estimates, it can efficiently perform Bayesian posterior sampling.

We evaluate sg-mcmc algorithms (Section 4) through an extensive experimental campaign, where we compare our approach to several alternatives, including Monte Carlo Dropout (mcd) [12] and Stochastic Weighted Averaging Gaussians (swag, [9]), which have been successfully applied to the Bayesian deep learning setting. Our results indicate that our approach offers performance that are competitive to the state of the art, according to metrics that aim at assessing the predictive accuracy and uncertainty.

## 2. Preliminaries and Related Work

Consider a dataset of *m*—dimensional observations $D={\left\{U_{i}\right\}}_{i=1}^{N}$. Given prior p(θ) for a *d*-dimensional set of parameters, and a likelihood model p(D|θ), the posterior is obtained by means of Bayes theorem as follows:

(1) $$p\left(\theta |D\right)=\frac{p(D|\theta )\;p(\theta )}{p(D)}$$

where p(D) is also known as the model evidence, defined as the integral p(D)=∫p(D|θ)p(θ)dθ. Except when the prior and the likelihood function are conjugate, Equation (1) is analytically intractable [13]. However, the joint likelihood term in the numerator is typically not hard to compute; this is a key element of many mcmc algorithms, since the normalization constant p(D) does not affect the shape of the distribution in any way other than scaling. The posterior distribution is necessary to obtain predictive distributions for new test observations $U_{*}$, as:

(2) $$p\left(U_{*}|D\right)=\int p\left(U_{*}|\theta \right)p\left(\theta |D\right)d\theta$$

We focus in particular on Monte Carlo methods to obtain an estimate of this predictive distribution, by averaging over $N_{\mathrm{MC}}$ samples obtained from the posterior over θ, i.e., $\theta^{\left(i\right)}∼p\left(\theta |D\right)$

(3) $$p\left(U_{*}|D\right)\approx \frac{1}{N_{\mathrm{MC}}}\sum_{i=1}^{N_{\mathrm{MC}}}p\left(U_{*}|\theta^{\left(i\right)}\right)$$

We develop our work by working with an unnormalized version of the logarithm of the posterior density, by expressing the negative logarithm of the joint distribution of the dataset D and parameters θ as:

(4) $$-f\left(\theta \right)=\sum_{i=1}^{N}\log p\left(U_{i}|\theta \right)+\log p\left(\theta \right).$$

For computational efficiency, we use a minibatch stochastic gradient g(θ), which guarantees that the estimated gradient is an unbiased estimate of the true gradient ∇f(θ), and we assume that the randomness due to the minibatch introduces a Gaussian noise:

(5) g(θ)∼N(∇f(θ),2B(θ)),

where the matrix B(θ) denotes the sg noise covariance, which depends on the parametric model, the data distribution and the minibatch size.

A survey of algorithms to sample from the posterior using sg methods can be found in Ma et al. [5]. In Appendix A we report some well-known facts which are relevant for the derivations in our paper. As shown in the literature [10,14], there are structural similarities between sg-mcmc algorithms and stochastic optimization methods, and both can be used to draw samples from posterior distributions. Notice that the original goal of stochastic optimization is to find the minimum of a given cost function, and the stochasticity is introduced by sub-sampling the dataset to scale. sg-mcmc methods instead aim at sampling from a given distribution, i.e., collecting multiple values, and the stochasticity is necessary explore the whole landscape. In what follows, we use a unified notation to compare many existing algorithms in light of the role played by their noise components.

It is well-known [15,16,17] that stochastic gradient descent (sgd), with and without momentum, can be studied through the following stochastic differential equation (sde), when the learning rate η is small enough (In this work we do not consider discretization errors. The reader can refer to classical sde texts such as [18] to investigate the topic in greater depth.):

(6) $$dz_{t}=s\left(z_{t}\right)dt+\sqrt{2\eta D(z_{t})}dW_{t}.$$

where s is usually referred to as driving force and D as diffusion matrix We use a generic form of the sde, with variable z instead of θ, which accommodates sgd variants, with and without momentum. By doing this, we will be able to easily cast the expression for the two cases in what follows (The operator $\nabla^{⊤}$ applied to matrix D(z) produces a row vector whose elements are the divergences of the D(z) columns. Our notation is aligned with Chen et al. [4]).

**Definition** **1.** *A distribution ρ(z)∝exp(−ϕ(z)) is said to be a* ***stationary*** *distribution for the* sde *of the form (6), if and only if it satisfies the following Fokker-Planck equation (* fpe *):*

(7) $$\begin{array}{c} 0=\mathrm{Tr}\left\{\nabla \left[-s{\left(z\right)}^{⊤}\rho \left(z\right)+\nabla^{⊤}\left(D\left(z\right)\rho \left(z\right)\right)\right]\right\}. \end{array}$$

Please note that in general, the stationary distribution does not converge to the desired posterior distribution, i.e., ϕ(z)≠f(z), as shown by Chaudhari and Soatto [8]. Additionally, given an initial condition for $z_{t}$, its distribution is going to converge to ρ(z) only for t→∞. In practice, we observe the sde dynamics for a finite amount of time: then, we declare that the process is approximately in the stationary regime once the potential has reached low and stable values.

Next, we briefly overview known approaches to Bayesian posterior sampling, and interpret them as variants of an sgd process, using the fpe formalism.

### 2.1. Gradient Methods without Momentum

The generalized updated rule of sgd, described as a discrete-time stochastic process, writes as:

(8) $$\begin{array}{c} \delta \theta_{n}=-\eta P\left(\theta_{n-1}\right)\left(g\left(\theta_{n-1}\right)+w_{n}\right), \end{array}$$

where $P(\theta_{n-1})$ is a user-defined preconditioning matrix, and $w_{n}$ is a noise term, distributed as $w_{n}∼N\left(0,2C\left(\theta_{n}\right)\right)$, with a user-defined covariance matrix $C(\theta_{n})$. Then, the corresponding continuous-time sde is [15]:

(9) $$d\theta_{t}=-P\left(\theta_{t}\right)\nabla f\left(\theta_{t}\right)dt+\sqrt{2\eta P{\left(\theta_{t}\right)}^{2}\sum \left(\theta_{t}\right)}dW_{t}.$$

In this paper we use the symbol *n* to indicate discrete time, while *t* for continuous time. We denote by C(θ) the covariance of the *injected noise* and ∑(θ) the *composite noise* covariance. Please note that $\sum \left(\theta_{t}\right)=B\left(\theta_{t}\right)+C\left(\theta_{t}\right)$ combines the sg and the injected noise. Notice that our choice of notation is different from the standard one, in which the starting discrete-time process is in the form $\delta \theta_{n}=-\eta P\left(\theta_{n-1}\right)\left(g\left(\theta_{n-1}\right)\right)+w_{n}$. By directly grouping the injected noise with the stochastic gradient we can better appreciate the relationship between annealing the learning rate and extremely large injected noise. Moreover, as will be explained in Section 3, this allows derivation of a new sampling algorithm.

We define the stationary distribution of the sde in Equation (9) as ρ(θ)∝exp(−ϕ(θ)). Please note that when C=0, the potential ϕ(θ) differs from the desired posterior f(θ) [8]. The following theorem, which is an adaptation of known results in light of our formalism, states the conditions for which the *noisy* sgd converges to the true posterior distribution (proof in Appendix A).

**Theorem** **1.** *Consider dynamics of the form (9) and define the stationary distribution ρ(θ)∝exp(−ϕ(θ)). If*

(10) $$\nabla^{⊤}\left(\sum {\left(\theta \right)}^{-1}\right)=0^{⊤}\;\;\;\;\mathrm{and}\;\;\;\;\eta P\left(\theta \right)=\sum {\left(\theta \right)}^{-1},$$

*then ϕ(θ)=f(θ).*

Stochastic Gradient Langevin Dynamics (sgld) [1] is a simple approach to satisfy Equation (10); it uses no preconditioning, P(θ)=I, and sets the injected noise covariance to $C\left(\theta \right)=\eta^{-1}I$. In the limit for η→0, it holds that $\sum \left(\theta \right)=B\left(\theta \right)+\eta^{-1}I≃\eta^{-1}I$. Then, $\nabla^{⊤}\left(\sum {\left(\theta \right)}^{-1}\right)=\eta \nabla^{⊤}I=0^{⊤}$, and $\eta P\left(\theta \right)=\sum {\left(\theta \right)}^{-1}$. Although sgld succeeds in (asymptotically) generating samples from the true posterior, its mixing rate is unnecessarily slow, due to the extremely small learning rate [2].

An extension to sgld is Stochastic Gradient Fisher Scoring (sgfs) [2], which can be tuned to switch between sampling from an approximate posterior, using a non-vanishing learning rate, and the true posterior, by annealing the learning rate to zero. sgfs uses preconditioning, $P\left(\theta \right)\propto B{\left(\theta \right)}^{-1}$. In practice, however, B(θ) is ill conditioned for complex models such as deep neural networks. Then, many of its eigenvalues are almost zero [8], and computing $B{\left(\theta \right)}^{-1}$ is problematic. An in-depth analysis of sgfs reveals that conditions (10) would be met with a non-vanishing learning rate only if, at convergence, $\nabla^{⊤}\left(B{\left(\theta \right)}^{-1}\right)=0^{⊤}$, which would be trivially true if B(θ) was constant. However, recent work [6,7] suggest that this condition is difficult to justify for deep neural networks.

The Stochastic Gradient Riemannian Langevin Dynamics (sgrld) algorithm [3] extends sgfs to the setting in which $\nabla^{⊤}\left(B{\left(\theta \right)}^{-1}\right)\neq 0^{⊤}$. The process dynamic is adjusted by adding the term $\nabla^{⊤}\left(B{\left(\theta \right)}^{-1}\right)$. However, the term $\nabla^{⊤}\left(B{\left(\theta \right)}^{-1}\right)$ has not a clear estimation procedure, restricting sgrld to cases where it can be computed analytically.

The work by [10] investigates constant-rate sgd (with no injected noise), and determines analytically the learning rate and preconditioning that minimize the Kullback–Leibler (kl) divergence between an approximation and the true posterior. Moreover, it shows that the preconditioning used in sgfs is optimal, in the sense that it converges to the true posterior, when B(θ) is constant and the true posterior has a quadratic form.

In summary, to claim convergence to the true posterior distribution, existing approaches require either vanishing learning rates or assumptions on the sg noise covariance that are difficult to verify in practice, especially when considering deep models. We instead propose a novel practical method that induces isotropic sg noise and thus satisfies Theorem 1. We determine analytically a fixed learning rate, and we require weaker assumptions on the loss shape.

### 2.2. Gradient Methods with Momentum

Momentum-corrected methods emerge as a natural extension to sgd approaches. The general set of update equations for (discrete-time) momentum-based algorithms is:

$$\begin{array}{c} \left\{\begin{array}{c} \delta \theta_{n}=\eta P\left(\theta_{n-1}\right)M^{-1}r_{n-1}\\ \delta r_{n}=-\eta A\left(\theta_{n-1}\right)M^{-1}r_{n-1}-\eta P\left(\theta_{n-1}\right)\left(g\left(\theta_{n-1}\right)+w_{n}\right), \end{array}\right. \end{array}$$

where $P(\theta_{n-1})$ is a preconditioning matrix, *M* is the mass matrix and $A(\theta_{n-1})$ is the friction matrix, as shown by [4,19]. As with the first order counterpart, the noise term is distributed as $w_{n}∼N\left(0,2C\left(\theta_{n}\right)\right)$. Then, the sde to describe continuous-time system dynamics is:

(11) $$\begin{array}{c} \left\{\begin{array}{c} d\theta_{t}=P\left(\theta_{t}\right)M^{-1}r_{t}dt\\ dr_{t}=-\left(A\left(\theta_{t}\right)M^{-1}r_{t}+P\left(\theta_{t}\right)\nabla f\left(\theta_{t}\right)\right)dt+\sqrt{2\eta P{\left(\theta_{t}\right)}^{2}\sum \left(\theta_{t}\right)}dW_{t}. \end{array}\right. \end{array}$$

where $P{\left(\theta_{t}\right)}^{2}=P\left(\theta_{t}\right)P\left(\theta_{t}\right)$ and we assume $P(\theta_{t})$ to be symmetric. The theorem hereafter describes the conditions for which noisy sgd with momentum converges to the true posterior distribution (Appendix A).

**Theorem** **2.** *Consider dynamics of the form (11) and define the stationary distribution for $\theta_{t}$ as ρ(θ)∝exp(−ϕ(θ)). If*

(12) $$\nabla^{⊤}P\left(\theta \right)=0^{⊤}\;\;\;\;\mathrm{and}\;\;\;\;A\left(\theta \right)=\eta P{\left(\theta \right)}^{2}\sum \left(\theta \right),$$

*then ϕ(θ)=f(θ).*

In the naive case, where P(θ)=I,A(θ)=0,C(θ)=0, Equation (12) are not satisfied and the stationary distribution does not correspond to the true posterior [4]. To generate samples from the true posterior it is sufficient to set P(θ)=I,A(θ)=ηB(θ),C(θ)=0 (as in Equation (9) in [4]).

Stochastic Gradient Hamiltonian Monte Carlo (sghmc) [4] suggests that estimating B(θ) can be costly. Hence, the injected noise C(θ) is chosen such that $C\left(\theta \right)=\eta^{-1}A\left(\theta \right)$, where A(θ) is user-defined. When η→0, the following approximation holds: ∑(θ)≃C(θ). It is then trivial to check that conditions (12) hold without the need for explicitly estimating B(θ). A further practical reason to avoid setting A(θ)=ηB(θ) is that the computational cost for the operation $A\left(\theta_{n-1}\right)M^{-1}r_{n-1}$ has $O(D^{2})$ complexity, whereas if C(θ) is diagonal, this is reduced to O(D). This, however, severely slows down the sampling process.

Stochastic Gradient Riemannian Hamiltonian Monte Carlo (sgrhmc) is an extension to sghmc [5]), which considers a generic, space-varying preconditioning matrix P(θ) derived from information geometric arguments [20]. sgrhmc suggests setting $P\left(\theta \right)=G{\left(\theta \right)}^{-\frac{1}{2}}$, where G(θ) is the Fisher Information matrix. To meet the requirement $\nabla^{⊤}P\left(\theta \right)=0^{⊤}$, it includes a correction term, $-\nabla^{⊤}P\left(\theta \right)$. The injected noise is set to $C\left(\theta \right)=\eta^{-1}I-B\left(\theta \right)$, consequently $\sum =\eta^{-1}I$, and the friction matrix is set to $A\left(\theta \right)=P{\left(\theta \right)}^{2}$. With all these choices, Theorem 2 is satisfied. Although appealing, the main drawbacks of this method are the need for an analytical expression of $\nabla^{⊤}P\left(\theta \right)$, and the assumption for B(θ) to be known.

From a practical standpoint, momentum-based methods suffer from the requirement to tune many hyperparameters, including the learning rate, and the parameters that govern the simulation of a second-order Langevin dynamics.

The method we propose in this work can be applied to momentum-based algorithms; in this case, it could be viewed as an extension of the work in [11], albeit addressing the complex loss landscapes typical of deep neural networks. However, we leave this avenue of research for future work.

## 3. Sampling by Layer-Wise Isotropization

We present a simple and practical approach to inject noise to SGD iterates to perform Bayesian posterior sampling. Our goal is to sample from the true posterior distribution (or approximations thereof) using a *constant* learning rate, and to rely on more lenient assumptions about the shape of the loss landscape that characterize deep models, compared to previous works. In general, in modern machine learning applications, we deal with multi-layer neural networks [21]. We exploit the natural subdivision of the parameters of these architecture into different layers to propose a practical sampling scheme

Careful inspection of Theorem 1 reveals that the matrices P(θ),∑(θ) are instrumental in determining the convergence properties of sg methods to the true posterior. Therefore, we consider the constructive approach of *designing* ηP(θ) to obtain a sampling scheme that meets our goals; we set ηP(θ) to be a constant, diagonal matrix which we constrain to be layer-wise uniform:

(13) $$\eta P\left(\theta \right)=\Lambda^{-1}=\mathrm{diag}{\left(\left[\underset{\mathrm{layer}\;1}{\underbrace{\lambda^{\left(1\right)},\ldots ,\lambda^{\left(1\right)}}},\ldots ,\underset{\mathrm{layer}\;N_{l}}{\underbrace{\lambda^{\left(N_{l}\right)},\ldots \lambda^{\left(N_{l}\right)}}}\right]\right)}^{-1}.$$

By properly selecting the set of parameters $\left\{\lambda^{i}\right\}$ we can achieve the simultaneous result of non-vanishing learning rate and well-conditioned preconditioning matrix. This implies a layer-wise learning rate $\eta^{\left(p\right)}=\frac{1}{\lambda^{\left(p\right)}}$ for the *p*-th layer, without further preconditioning.

We can now prove (see Appendix B), as a corollary to Theorem 1, that our design choices can guarantee convergence to the true posterior distribution.

**Corollary** **1.** *(Theorem 1) Consider dynamics of the form (9) and define the stationary distribution ρ(θ)∝exp(−ϕ(θ)). If $\eta P\left(\theta \right)=\Lambda^{-1}$ as in (13), C(θ)=Λ−B(θ) and C(θ)≻0∀θ, then ϕ(θ)=f(θ).*

If aforementioned conditions are satisfied, it is in fact simple to show that the relevant matrices satisfy the conditions in Equation (10). The covariance matrix of the composite noise is said to be *isotropic* within the layers of (deep) models. In fact, $\sum \left(\theta \right)=C\left(\theta \right)+B\left(\theta \right)=\mathrm{diag}\left(\left[\begin{array}{c} \lambda^{\left(1\right)},\ldots ,\lambda^{\left(1\right)},\ldots ,\lambda^{\left(N_{l}\right)},\ldots \lambda^{\left(N_{l}\right)} \end{array}\right]\right)$. From a practical point of view, we choose Λ to be, among all valid matrices satisfying Λ−B(θ)≻0, the smallest (the one with the smallest λ’s). Indeed, larger Λ induce a smaller learning rate, thus unnecessarily reducing sampling speed.

Now, let us consider an ideal case, in which we assume the sg noise covariance B(θ) and Λ to be known in advance. The procedure described in Algorithm 1 illustrates a naive sg method that uses the *injected noise* covariance C(θ) to sample from the true posterior.
**Algorithm 1** Idealized posterior sampling {Initialization: $\theta_{0}$}$\mathrm{SAMPLE}\;(\theta_{0},\;B(\theta ),\;\Lambda ):$$\theta \;\leftarrow \;\theta_{0}$ **loop**  $g\;=\;\nabla \tilde{f}(\theta )$  n ∼ N(0,I)  $C{\left(\theta \right)}^{1/2}\;\leftarrow \;{\left(\sum \;-\;B(\theta )\right)}^{1/2}$  $g\;\leftarrow \;\sum^{-1}(g+\sqrt{2}C{\left(\theta \right)}^{1/2}n)$  θ ← θ−g **end**
**loop**

This deceivingly simple procedure generate samples from the true posterior, with a non-vanishing learning rate, as shown earlier. However, it cannot be used in practice as B(θ) and Λ are unknown. Furthermore, the algorithm requires computationally expensive operations, i.e., to compute ${\left(\sum -B\left(\theta \right)\right)}^{\frac{1}{2}}$, which requires $O(d^{3})$ operations, and $C{\left(\theta \right)}^{\frac{1}{2}}$, which costs $O(d^{2})$ multiplications.

Next, we describe a practical variant of our approach, where we use approximations at the expense of generating samples from the true posterior distribution. We note that [10] suggest exploring a related preconditioning, but do not develop this path in their work. Moreover, the proposed method shares similarities with a scheme proposed in [22] although the analysis we perform here is different.

### 3.1. A Practical Method: Isotropic SGD

To render the idealized sampling method practical, it is necessary to consider some additional assumptions. As we explain at the end of this section, the assumptions that follow are less strict than other approaches in the literature.

**Assumption** **1.** *The* sg *noise covariance B(θ) can be approximated with a diagonal matrix, i.e., B(θ)=diag(b(θ)).*

**Assumption** **2.** *The signal-to-noise ratio (SNR) of a gradient is small enough such that in the stationary regime, the second-order moment of the gradient is a good estimate of the true variance. Hence, combining with Assumption 1, $b\left(\theta \right)≃\frac{E[g(\theta )⊙g(\theta )]}{2}$, where* ⊙ *indicates the elementwise product.*

**Assumption** **3.** *The sum of the variances of noise components, layer by layer, can be assumed to constant in the stationary regime. Then, $\beta^{\left(p\right)}=\sum_{j\in I_{p}}b_{j}\left(\theta \right)$, where $I_{p}$ is the set of indices of parameters belonging to $p_{th}$ layer.*

The diagonal covariance assumption (i.e., Assumption 1) is common in other works, such as [2,11]. The small signal-to-noise ratio as stated in Assumption 2 is in line with recent studies, such as [11,23]. Assumption 3 is similar to those appeared in earlier work, such as [24]. Please note that Assumptions 2 and 3 must hold in the stationary regime when the process reaches the bottom valley of the loss landscape. The matrix (b(θ)) has been associated in the literature with the *empirical* Fisher information matrix [2,25]. As we do not consider this matrix for preconditioning purposes, we do not further investigate this connection.

Given our assumptions, and our design choices, it is then possible to show (see Appendix B) that the optimal (i.e., the smallest possible) $\Lambda =\left[\begin{array}{c} \lambda^{\left(1\right)},\ldots ,\lambda^{\left(1\right)},\ldots ,\lambda^{\left(N_{l}\right)},\ldots \lambda^{\left(N_{l}\right)} \end{array}\right]$ satisfying Corollary 1 can be obtained as $\lambda^{\left(p\right)}=\beta^{\left(p\right)}$. Please note that we do not assume B(θ) to be known, but use a simple procedure to estimate its components by computing: $\lambda^{\left(p\right)}=\sum_{j\in I_{p}}b_{j}\left(\theta \right)=\frac{\left|\right|g^{\left(p\right)}\left(\theta \right){\left|\right|}^{2}}{2},$ where $g^{\left(p\right)}\left(\theta \right)$ is the portion of stochastic gradient corresponding to the *p*-th layer. Then, the composite noise matrix ∑=Λ is a layer-wise isotropic covariance matrix, which inspires the name of our proposed method as *Isotropic* SGD (i-sgd).

The practical implementation of i-sgd is shown in Algorithm 2. The advantage of i-sgd is that it can either be used to obtain posterior samples starting from a pre-trained model, or do so by training a model from scratch. In either case, the estimates of B(θ) are used to compute Λ, as discussed above. An important consideration is that once all $\lambda^{\left(i\right)}$ have been estimated, the learning rate, layer by layer, is determined *automatically*. In fact, for the *p*-th layer, the learning rate is: $\eta^{\left(p\right)}={\lambda^{\left(p\right)}}^{-1}$. A simpler approach is to use a unique learning rate for all layers, where the equivalent λ is the sum of all $\lambda^{\left(p\right)}$.
**Algorithm 2**i-sgd: practical posterior sampling$\mathrm{SAMPLE}\;(\theta_{0}):$$\theta \;\leftarrow \;\theta_{0}$ **loop**  $g\;=\;\nabla \tilde{f}(\theta )$  **for**
p ← 1 to *N_l_*
**do**   n ∼ N(0,I)   $C{\left(\theta \right)}^{1/2}\;\leftarrow \;(\lambda^{\left(p\right)}\;-\;(1/2)\;(g^{\left(p\right)}⊙g^{\left(p\right)}))$   $g^{\left(p\right)}\;\leftarrow \;1/\lambda^{\left(p\right)}(g^{\left(p\right)}\;+\;\sqrt{2}C{\left(\theta \right)}^{1/2}n)$  **end**
**for**  θ ← θ−g **end**
**loop**

#### A Remark on Convergence

In summary, i-sgd is a practical method to perform approximate Bayesian posterior sampling, backed up by solid theoretical foundations. Our assumptions, which are at the origin of the approximate nature of i-sgd, are less strict than those used in the literature of sg-mcmc methods. More precisely, the theory behind i-sgd can explain convergence to the true posterior with a non-vanishing learning rate in the particular case when Assumption 1 holds and the estimation of B(θ) is perfect. Even with perfect estimates, this is not the case for sgfs, which requires the correction term $\nabla^{⊤}B{\left(\theta \right)}^{-1}=0$. Additionally, both sgrld and sgrhmc are more demanding than i-sgd because they require computing $\nabla^{⊤}B{\left(\theta \right)}^{-1}$, for which an estimation procedure is elusive. Finally, the method by Springenberg et al. [11] needs a *constant*, diagonal B(θ), a condition that does not necessarily hold for deep models.

### 3.2. Computational Cost

The computational cost of i-sgd is as follows. As with [4], we define the cost of computing a gradient minibatch as $C_{g}\left(N_{b},d\right)$. Thanks to Assumptions 1 and 2, the computational cost for estimating the noise covariance scales as O(d) multiplications. The computational cost of generating random samples with the desired covariance scales as O(d) square roots and O(d) multiplications (without considering the cost of generating random numbers). The overall cost of our method is the sum of the above terms. Notice that the cost of estimating the noise covariance does not depend on the minibatch size $N_{b}$. We would like to stress that in many modern models, the real computational bottleneck is the backward propagation for the computation of the gradients. As all the sg-mcmc methods considered in this work require one gradient evaluation per step, the different methods have in practice the same complexity.

The space complexity of i-sgd is the same as sghmc,sgfs and variants: it scales as $O(N_{\mathrm{sam}}d)$, where $N_{\mathrm{sam}}$ is the number of posterior samples.

## 4. Experiments

The empirical analysis of our method, and its comparison to alternative approaches from the literature, is organized as follows. First, we proceed with a validation of i-sgd using the standard uci datasets [26] and a shallow neural network. Then we move to the case of deeper models: we begin with a simple CNN used on the mnist [27] dataset, then move to the standard ResNet-18 [28] deep network using the Cifar-10 [29] dataset.

We compare i-sgd to other Bayesian sampling methods such as sghmc [4], sgld [2], and to alternative approaches to approximate Bayesian inference, including mcd [12], swag [9] and vsgd [10]. In general, our result indicates that: (1) i-sgd achieves similar or superior performance regarding competitors, when measuring uncertainty quantification, even with simple datasets and models; (2) i-sgd is simple to tune, when compared to alternatives; (3) i-sgd is competitive when used for deep Bayesian modeling, even when compared to standard methods used in the literature. In particular, the proposed method shares some of the strengths of vsgd, such as learning rates determined automatically and the simplicity of sgld. Appendix B includes additional implementation details on i-sgd. Appendix C presents detailed configurations of all methods we compare, and additional experimental results.

### 4.1. A Disclaimer on Performance Characterization

It is important to stress a detail on the analysis of the experimental campaign. The discussion is usually focused on the goodness of the various methods for representing the true posterior distribution. Different methods can or cannot claim convergence to the true posterior according to certain assumptions and the nature of the hyperparameters. In the experimental validation of the results, however, we do not have access to the form of the true posterior as it is exactly the problem we are trying to solve. The practical solution adopted is to compare the different methods in terms of *proxy* metrics evaluated on the test sets, such as the accuracy and uncertainty metrics. Being better in terms of these performance metrics does not imply that the sampling method is better at approximating the posterior distribution, and outperforming competitors in terms of these metric do not provide sufficient information about the intrinsic quality of the sampling scheme.

### 4.2. Regression Tasks, with Simple Models

We consider several regression tasks defined on the uci datasets. We use a simple neural network configuration with two fully connected layers and a relu activation function; the hidden layer includes 50 units. In this set of experiments, we use the following metrics: the root mean square error (rmse) to judge the model predictive performance and the mean negative log-likelihood (mnll) as a proxy for uncertainty quantification. We note that the task of tuning our competitors was far from trivial. We used our own version of sghmc, based on [11], to ensure a proper understanding of the implementation internals, and we proceeded with a tuning process to find appropriate values for the numerous hyperparameters. In this set of experiments, we omit results for swag, which we keep for more involved scenarios.

Table 1 and Table 2 report a complete overview of our results, for a selection of uci datasets. For each method and each dataset, we also included how many out of the 10 splits considered failed to converge, indicated as F=…. As explained in Appendix C we implemented a temperature scaled version of vsgd. A clear picture emerges from this first set of experiments: while for the rmse the performance is similar for different methods, for the mnll averaging over multiple samples clearly improves the uncertainty quantification capabilities. sghmc is in many cases better than alternatives, considering however the standard deviation of the results it is difficult to claim clear superiority of one method over the others.

### 4.3. Classification Tasks, with Deeper Models

Next, we compare i-sgd against competitors on image classification tasks. First, we use the mnist dataset, and a simple LeNet-5 CNN [30]. All methods are compared based on the test accuracy acc,mnll and the expected calibration error (ece, 31]). Additionally, at test time, we carry out predictions on both mnist and not-mnist; the latter is a dataset equivalent to mnist, but it represents letters rather than numbers. (http://yaroslavvb.blogspot.com/2011/09/notmnist-dataset.html, accessed on 24 October 2021) This experimental setup is often used to check whether the entropy of the predictions on not-mnist is higher than the entropy of the predictions on mnist (the entropy of the output of an $N_{cl}$ classes classifier, represented by the vector p, is defined as $-\sum_{i=1}^{N_{cl}}p_{i}\log p_{i}$).

**Figure 1:**
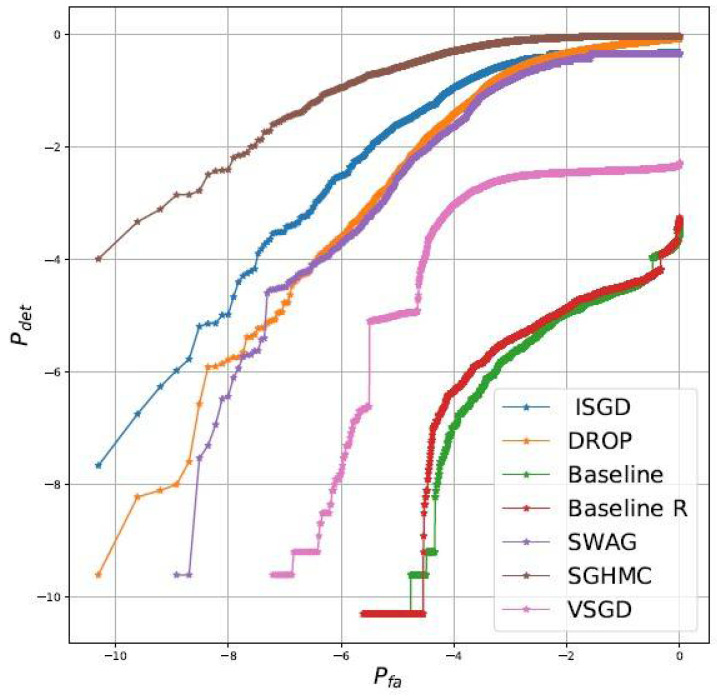
Detection/False alarm diagrams for different methods.

Table 3 indicates that all methods are essentially equivalent in terms of accuracy and mnll. We consider, together with the classical in and out of distribution entropies the regions of convergence (rocs) diagrams comparing detection of out of distribution samples and false alarms when using as test statistic the entropy. Results, reported in Figure 1, clearly shows that: (1) collecting multiple samples improve the uncertainty quantification capabilities (2) i-sgd is competitive (but not the best scheme) and importantly outperform the closest approach to ours, i.e., vsgd. The experimental results show that i-sgd improves the quality of the baseline model with respect to all metrics. To test whether the improvements are due just to “*additional training*” or are intrinsically due to the Bayesian averaging properties, we do consider alternative deterministic baselines (details in Appendix C). For this set of experiments the best performing is baseline R. As can be appreciated by comparing Table 3 and Figure 1, while it is possible to increase the classical metrics, i-sgd (and other methods) still outperform by a large margin the baselines in terms of detection of out of distribution samples.

We now move on to a classical image classification problem with deep convolutional networks, whereby we use the cifar10 dataset, and the ResNet-18 network architecture. For this set of experiments, we compare i-sgd, sghmc, swag, and vsgd using again test accuracy and mnll, which we report in Table 4. As usual, we compare the results against the baseline of the individual network resulting from the pre-training phase. Results are obtained averaging over three independent seeds. Notice, as expanded in Appendix C that for swag we do consider two variants: the Bayesian correct one (swag) and a second variant that has better performance (swag wd). We stress again, as highlighted in Section 4.1 that not always goodness of approximation of the posterior and performance correlate positively. Additionally in this case, we found i-sgd to be competitive with other methods and superior to the baseline. Among the competitors, we found i-sgd to the easiest to tune, given the feature of a fixed learning rate informed by theoretical considerations; we believe that this is an important aspect to consider for a wide adoption of our proposal by practitioners.

## 5. Conclusions

sg methods allowed Bayesian posterior sampling algorithms, such as mcmc, to regain relevance in an age when datasets have reached extremely large sizes. However, despite mathematical elegance and promising results, current approaches from the literature are restricted to simple models. Indeed, the sampling properties of these algorithms are determined by simplifying assumptions on the loss landscape, which do not hold for the kind of complex models which are popular these days, such as deep models. Meanwhile, sg-mcmc algorithms require vanishing learning rates, which force practitioners to develop creative annealing schedules that are often model specific and difficult to justify.

We have attempted to target these weaknesses by suggesting a simpler algorithm that relies on fewer parameters and less strict assumptions compared to the literature on sg-mcmc. We used a unified mathematical notation to deepen our understanding of the role of the covariance of the noise of stochastic gradients and learning rate on the behavior of sg-mcmc algorithms. We then presented a practical variant of the sgd algorithm, which uses a constant learning rate, and an additional noise to perform Bayesian posterior sampling. Our proposal is derived from the ideal method, in which it is guaranteed that samples are generated from the true posterior. When the learning rate and noise terms are empirically estimated, with no user intervention, our method offers a very good approximation to the posterior, as demonstrated by the extensive experimental campaign.

We verified empirically the quality of our approach, and compared its performance to state-of-the-art sg-mcmc and alternative methods. Results, which span a variety of settings, indicated that our method is competitive to the alternatives from the state-of-the-art, while being much simpler to use.

## Figures and Tables

**Table 1:** rmse results for regression on uci datasets.

Method	wine	protein	naval	kin8nm	power	boston
**sgld**	0.759 ± 0.07	5.687 ± 0.05	0.007 ± 0.00 (F = 6.000)	0.171 ± 0.07 (F = 3.000)	11.753 ± 3.25	9.602 ± 2.06
**i-sgd**	0.635 ± 0.05	4.699 ± 0.03	0.001 ± 0.00	0.079 ± 0.00	4.320 ± 0.13	3.703 ± 1.19
**Baseline**	0.641 ± 0.05	4.733 ± 0.05	0.001 ± 0.00	0.080 ± 0.00	4.354 ± 0.12	3.705 ± 1.19
**vsgd**	0.635 ± 0.05	4.699 ± 0.03	0.001 ± 0.00	0.079 ± 0.00	4.325 ± 0.13	3.588 ± 1.06 (F = 1.000)
**sghmc**	0.628 ± 0.04	4.712 ± 0.03	0.000 ± 0.00 (F = 2.000)	0.076 ± 0.00 (F = 1.000)	4.310 ± 0.14	3.659 ± 1.24
**sgld** T	0.752 ± 0.07	5.673 ± 0.04	0.007 ± 0.00 (F = 6.000)	0.169 ± 0.07 (F = 3.000)	11.351 ± 3.02	9.417 ± 2.07
**drop**	0.637 ± 0.04	4.968 ± 0.05	0.003 ± 0.00	0.139 ± 0.01	4.531 ± 0.16	3.803 ± 1.26
**sghmc** T	0.628 ± 0.04	4.684 ± 0.03	0.000 ± 0.00 (F = 6.000)	0.076 ± 0.00	4.326 ± 0.13	3.692 ± 1.19

**Table 2:** mnll results for regression on uci datasets.

Method	wine	protein	naval	kin8nm	power	boston
**sgld**	1.546 ± 0.25	5.604 ± 0.08	−1.751 ± 0.28 (F = 6.000)	5.140 ± 7.05 (F=3.000)	8.429 ± 3.14	30.386 ± 15.77
**i-sgd**	1.129 ± 0.15	4.371 ± 0.03	−2.466 ± 1.12	−0.460 ± 0.65	3.122 ± 0.07	9.799 ± 5.69
**Baseline**	1.182 ± 0.03	3.964 ± 0.04	0.920 ± 0.00	0.924 ± 0.00	3.071 ± 0.06	5.421 ± 2.73
**vsgd**	1.128 ± 0.15	4.371 ± 0.03	−2.466 ± 1.12	−0.480 ± 0.65	3.088 ± 0.06	8.413 ± 5.89 (F = 1.000)
**sghmc**	1.041 ± 0.12	4.142 ± 0.02	−2.763 ± 1.33 (F = 2.000)	−0.798 ± 0.39 (F = 1.000)	2.924 ± 0.04	3.097 ± 0.83
**sgld** T	1.526 ± 0.24	5.591 ± 0.07	−1.752 ± 0.28 (F = 6.000)	5.118 ± 7.06 (F = 3.000)	8.288 ± 3.04	33.212 ± 19.69
**drop**	1.065 ± 0.12	4.218 ± 0.06	−2.322 ± 0.75	−0.086 ± 0.41	2.941 ± 0.04	3.989 ± 1.23
**sghmc** T	1.104 ± 0.14	4.191 ± 0.02	−2.966 ± 1.89 (F = 6.000)	−0.756 ± 0.42	3.116 ± 0.07	9.826 ± 5.72

**Table 3:** Results for classification on mnist dataset.

Method	acc	mnll	Mean $H_{0}$	ece	Mean $H_{1}$	Failed
i-sgd	9916.3333 ± 2.8674	263.5311 ± 16.3600	0.0368 ± 0.0019	0.0491 ± 0.0003	0.4558 ± 0.0591	0.0000
sghmc	9930.6667 ± 2.4944	268.2559 ± 6.8172	0.0593 ± 0.0018	0.0531 ± 0.0003	1.0369 ± 0.0346	0.0000
drop	9912.6667 ± 6.0185	362.8973 ± 24.8881	0.0960 ± 0.0090	0.0541 ± 0.0011	0.5507 ± 0.0577	0.0000
baseline	9886.6667 ± 11.0252	352.6640 ± 20.8622	0.0353 ± 0.0058	0.0468 ± 0.0001	0.0019 ± 0.0003	0.0000
baseline r	9919.0000 ± 9.4163	242.7644 ± 17.0736	0.0303 ± 0.0001	0.0482 ± 0.0006	0.0021 ± 0.0002	0.0000
swag	9917.0000 ± 2.8284	308.8182 ± 20.0979	0.0675 ± 0.0108	0.0524 ± 0.0011	0.3953 ± 0.0442	0.0000
sgld	9927.0000 ± 1.0000	279.7685 ± 16.6563	0.0556 ± 0.0034	0.0531 ± 0.0004	1.3032 ± 0.1942	1.0000
vsgd	9927.3333 ± 6.7987	225.3725 ± 16.3739	0.0274 ± 0.0008	0.0481 ± 0.0005	0.0414 ± 0.0070	0.0000
i-sgd T	9915.6667 ± 0.9428	255.9641 ± 12.8051	0.0289 ± 0.0014	0.0478 ± 0.0002	0.0284 ± 0.0122	0.0000
sghmc T	9937.0000 ± 0.0000	231.5332 ± 0.0000	0.0434 ± 0.0000	0.0518 ± 0.0000	0.4623 ± 0.0000	2.0000

**Table 4:** Results for classification on cifar1010 dataset.

Method	acc	mnll	mean $H_{0}$	ece
i-sgd	8591.3333 ± 17.4611	4393.3557 ± 107.0878	0.6107 ± 0.0337	0.0731 ± 0.0075
sghmc	8634.6667 ± 5.1854	4357.8998 ± 11.2722	0.6300 ± 0.0023	0.0819 ± 0.0017
swag wd	8740.6667 ± 35.5653	3931.9900 ± 45.6605	0.4130 ± 0.0066	0.0275 ± 0.0015
swag	8061.0000 ± 11.4310	5903.2605 ± 62.8167	0.5308 ± 0.0135	0.0163 ± 0.0019
baseline	8273.3333 ± 26.7872	8050.4467 ± 109.9864	0.2250 ± 0.0005	0.0809 ± 0.0020
vsgd	8255.6667 ± 24.1155	8919.8062 ± 106.3571	0.1761 ± 0.0078	0.0905 ± 0.0020

## Data Availability

Not applicable.

## Appendix A. Background and Related Material

### Appendix A.1. The Minibatch Gradient Approximation

Starting from the gradient of the logarithm of the posterior density:

$$-\nabla f\left(\theta \right)=\sum_{i=1}^{N}\nabla \log p\left(U_{i}|\theta \right)+\nabla \log p\left(\theta \right),$$

it is possible to define its *minibatch* version by computing the gradient on a random subset $I_{N_{b}}$ with cardinality $N_{b}$ of all the indices. The minibatch gradient g(θ) is computed as

$$-g\left(\theta \right)=\frac{N}{N_{b}}\sum_{i=1}^{N_{b}}\nabla \log p\left(U_{i}|\theta \right)+\nabla \log p\left(\theta \right),$$

By simple calculations it is possible to show that the estimation is unbiased (E(g(θ))=∇f(θ)). The estimation error covariance is defined to be $E\left[\left(g(\theta )-\nabla f(\theta )\right){\left(g(\theta )-\nabla f(\theta )\right)}^{⊤}\right]=2B\left(\theta \right)$.

If the minibatch size is large enough, invoking the central limit theorem, we can state that the minibatch gradient is normally distributed:

g(θ)∼N(∇f(θ),2B(θ)).

### Appendix A.2. Gradient Methods without Momentum

#### Appendix A.2.1. The sde from Discrete Time

We start from the generalized updated rule of sgd:

$$\begin{array}{c} \delta \theta_{n}=-\eta P\left(\theta_{n-1}\right)\left(g\left(\theta_{n-1}\right)+w_{n}\right). \end{array}$$

Since $g\left(\theta_{n-1}\right)∼N\left(\nabla f\left(\theta_{n-1}\right),2B\left(\theta_{n-1}\right)\right)$ we can rewrite the above equation as:

$$\begin{array}{c} \delta \theta_{n}=-\eta P\left(\theta_{n-1}\right)\left(\nabla f\left(\theta_{n-1}\right)+w_{n}^{{}^{\prime }}\right), \end{array}$$

where $w_{n}^{{}^{\prime }}∼N\left(0,2\sum \left(\theta_{n-1}\right)\right)$. If we separate deterministic and random component we can equivalently write:

$$\begin{array}{cc} & \delta \theta_{n}=-\eta P\left(\theta_{n-1}\right)\nabla f\left(\theta_{n-1}\right)+\eta P\left(\theta_{n-1}\right)w_{n}^{{}^{\prime }}=-\eta P\left(\theta_{n-1}\right)\nabla f\left(\theta_{n-1}\right)+\\ & \sqrt{2\eta P^{2}\left(\theta_{n-1}\right)\sum \left(\theta_{n-1}\right)}v_{n} \end{array}$$

where $v_{n}∼N\left(0,\sqrt{\eta }I\right)$. When η is small enough (η→dt) we can interpret the above equation as the discrete-time simulation of the following sde [15]:

$$d\theta_{t}=-P\left(\theta_{t}\right)\nabla f\left(\theta_{t}\right)dt+\sqrt{2\eta P{\left(\theta_{t}\right)}^{2}\sum \left(\theta_{t}\right)}dW_{t},$$

where $dW_{t}$ is a *d*—dimensional Brownian motion.

#### Appendix A.2.2. Proof of Theorem 1

The stationary distribution of the above sde, ρ(θ)∝exp(−ϕ(θ)), satisfies the following fpe

$$\begin{array}{c} 0=\mathrm{Tr}\left\{\nabla \left[\nabla^{⊤}\left(f(\theta )\right)P\left(\theta \right)\rho \left(\theta \right)+\eta \nabla^{⊤}\left(P{\left(\theta \right)}^{2}\sum \left(\theta \right)\rho \left(\theta \right)\right)\right]\right\}, \end{array}$$

that we rewrite as

$$\begin{array}{c} 0=\mathrm{Tr}\{\nabla \left[\nabla^{⊤}\left(f(\theta )\right)P\left(\theta \right)\rho \left(\theta \right)-\eta \nabla^{⊤}\left(\phi \left(\theta \right)\right)P{\left(\theta \right)}^{2}\sum \left(\theta \right)\rho \left(\theta \right)+\eta \nabla^{⊤}\left(P{\left(\theta \right)}^{2}\sum \left(\theta \right)\right)\rho \left(\theta \right)\right]\}. \end{array}$$

The above equation is verified with ∇f(θ)=∇ϕ(θ) if

$$\left\{\begin{array}{c} \nabla^{⊤}\left(P{\left(\theta \right)}^{2}\sum \left(\theta \right)\right)=0\\ \eta P{\left(\theta \right)}^{2}\sum \left(\theta \right)=P\left(\theta \right)\to \eta P\left(\theta \right)=\sum {\left(\theta \right)}^{-1} \end{array}\right.$$

that proves Theorem 1.

### Appendix A.3. Gradient Methods with Momentum

#### Appendix A.3.1. The SDE from Discrete Time

The general set of update equations for (discrete-time) momentum-based algorithms is:

$$\begin{array}{c} \left\{\begin{array}{c} \delta \theta_{n}=\eta P\left(\theta_{n-1}\right)M^{-1}r_{n-1}\\ \delta r_{n}=-\eta A\left(\theta_{n-1}\right)M^{-1}r_{n-1}-\eta P\left(\theta_{n-1}\right)\left(g\left(\theta_{n-1}\right)+w_{n}\right). \end{array}\right. \end{array}$$

Similarly to the case without momentum, we rewrite the second equation of the system as

$$\begin{array}{cc} & \delta r_{n}=-\eta A\left(\theta_{n-1}\right)M^{-1}r_{n-1}-\eta P\left(\theta_{n-1}\right)\left(g\left(\theta_{n-1}\right)+w_{n}\right)=\\ & -\eta A\left(\theta_{n-1}\right)M^{-1}r_{n-1}-\eta P\left(\theta_{n-1}\right)\nabla f\left(\theta_{n-1}\right)+\sqrt{2\eta P^{2}\left(\theta_{n-1}\right)\sum \left(\theta_{n-1}\right)}v_{n} \end{array}$$

where again $v_{n}∼N\left(0,\sqrt{\eta }I\right)$. If we define the supervariable $z={\left[\theta ,r\right]}^{⊤}$ we can rewrite the system as

$$\begin{array}{c} \delta z_{n}=-\eta \left[\begin{array}{cc} 0 & -P(\theta_{n-1})\\ P(\theta_{n-1}) & A(\theta_{n-1}) \end{array}\right]s\left(z_{n-1}\right)+\sqrt{2\eta D(z_{n-1})}\nu_{n} \end{array}$$

where $s\left(z\right)=\left[\begin{array}{c} \nabla f(\theta )\\ M^{-1}r \end{array}\right]$, $D\left(z\right)=\left[\begin{array}{cc} 0 & 0\\ 0 & P{\left(\theta \right)}^{2}\sum \left(\theta \right) \end{array}\right]$ and $\nu_{n}∼N\left(0,\sqrt{\eta }I\right)$.

As the learning rate goes to zero (η→dt), similarly to the previous case, we can interpret the above difference equation as a discretization of the following fpe

$$\begin{array}{c} dz_{t}=-\left[\begin{array}{cc} 0 & -P(\theta_{t})\\ P(\theta_{t}) & A(\theta_{t}) \end{array}\right]s\left(z_{t}\right)+\sqrt{2\eta D(z_{t})}dW_{t} \end{array}$$

#### Appendix A.3.2. Proof of Theorem 2

As before we assume that the stationary distribution has form ρ(z)∝exp(−ϕ(z)). The corresponding fpe is

$$\begin{array}{c} 0=\mathrm{Tr}\left(\nabla \left(s{\left(z\right)}^{⊤}\left[\begin{array}{cc} 0 & -P(\theta )\\ P(\theta ) & A(\theta ) \end{array}\right]\rho \left(z\right)+\eta \left(\nabla^{⊤}\left(D(z)\rho (z)\right)\right)\right)\right). \end{array}$$

Notice that since $\nabla^{⊤}D\left(z\right)=0$ we can rewrite

$$\begin{array}{cc} & 0=\mathrm{Tr}\left(\nabla \left(s{\left(z\right)}^{⊤}\left[\begin{array}{cc} 0 & -P(\theta )\\ P(\theta ) & A(\theta ) \end{array}\right]\rho \left(z\right)+\eta \nabla^{⊤}\left(\rho \left(z\right)\right)D\left(z\right)\right)\right)\\ & =\mathrm{Tr}\left(\nabla \left(s{\left(z\right)}^{⊤}\left[\begin{array}{cc} 0 & -P(\theta )\\ P(\theta ) & A(\theta ) \end{array}\right]\rho \left(z\right)-\eta \nabla^{⊤}\left(\phi \left(z\right)\right)D\left(z\right)\rho \left(z\right)\right)\right)\\ & =\mathrm{Tr}\left(\nabla \left(s{\left(z\right)}^{⊤}\left[\begin{array}{cc} 0 & -P(\theta )\\ P(\theta ) & A(\theta ) \end{array}\right]\rho \left(z\right)-\eta \nabla^{⊤}\left(\phi \left(z\right)\right)\left[\begin{array}{cc} 0 & 0\\ 0 & P{\left(\theta \right)}^{2}\sum \left(\theta \right) \end{array}\right]\rho \left(z\right)\right)\right) \end{array}$$

that is verified with ∇ϕ(z)=s(z) if

$$\left\{\begin{array}{c} \nabla^{⊤}P\left(\theta \right)=0\\ A\left(\theta \right)=\eta P{\left(\theta \right)}^{2}\sum \left(\theta \right). \end{array}\right.$$

If $\nabla^{⊤}P\left(\theta \right)=0$ in fact

$$\begin{array}{cc} & \mathrm{Tr}\left(\nabla \left(\nabla^{⊤}\left(\phi \left(z\right)\right)\rho \left(z\right)\left[\begin{array}{cc} 0 & -P(\theta )\\ P(\theta ) & 0 \end{array}\right]\right)\right)=\nabla^{⊤}\left(\left[\begin{array}{cc} 0 & -P(\theta )\\ P(\theta ) & 0 \end{array}\right]\nabla \left(\phi \left(z\right)\right)\rho \left(z\right)\right)=\\ & \nabla^{⊤}\left(\left[\begin{array}{cc} 0 & -P(\theta )\\ P(\theta ) & 0 \end{array}\right]\right)\nabla \left(\phi \left(z\right)\right)\rho \left(z\right)+\mathrm{Tr}\left(\left[\begin{array}{cc} 0 & -P(\theta )\\ P(\theta ) & 0 \end{array}\right]\nabla \left(\nabla^{⊤}\left(\phi \left(z\right)\right)\rho \left(z\right)\right)\right)=0, \end{array}$$

since $\nabla^{⊤}\left[\begin{array}{cc} 0 & -P(\theta )\\ P(\theta ) & 0 \end{array}\right]=0$ and the second term is zero due to the fact that $\left[\begin{array}{cc} 0 & -P(\theta )\\ P(\theta ) & 0 \end{array}\right]$ is anti-symmetric while $\nabla \left(\nabla^{⊤}\left(\phi \left(z\right)\right)\rho \left(z\right)\right)$ is symmetric.

Thus, we can rewrite

$$\begin{array}{cc} & \mathrm{Tr}\left(\nabla \left(s{\left(z\right)}^{⊤}\left[\begin{array}{cc} 0 & -P(\theta )\\ P(\theta ) & A(\theta ) \end{array}\right]\rho \left(z\right)-\eta \nabla^{⊤}\left(\phi \left(z\right)\right)\left[\begin{array}{cc} 0 & 0\\ 0 & P{\left(\theta \right)}^{2}\sum \left(\theta \right) \end{array}\right]\rho \left(z\right)\right)\right)=\\ & \mathrm{Tr}\left(\nabla \left(s{\left(z\right)}^{⊤}\left[\begin{array}{cc} 0 & -P(\theta )\\ P(\theta ) & A(\theta ) \end{array}\right]\rho \left(z\right)-\nabla^{⊤}\left(\phi \left(z\right)\right)\left[\begin{array}{cc} 0 & 0\\ 0 & \eta P{\left(\theta \right)}^{2}\sum \left(\theta \right) \end{array}\right]\rho \left(z\right)\right)\right)=\\ & \mathrm{Tr}\left(\nabla \left(s{\left(z\right)}^{⊤}\left[\begin{array}{cc} 0 & -P(\theta )\\ P(\theta ) & A(\theta ) \end{array}\right]\rho \left(z\right)-\nabla^{⊤}\left(\phi \left(z\right)\right)\left[\begin{array}{cc} 0 & 0\\ 0 & A(\theta ) \end{array}\right]\rho \left(z\right)\right)\right)=\\ & \mathrm{Tr}\left(\nabla \left(\left(s{\left(z\right)}^{⊤}-\nabla^{⊤}\left(\phi \left(z\right)\right)\right)\left[\begin{array}{cc} 0 & -P(\theta )\\ P(\theta ) & A(\theta ) \end{array}\right]\rho \left(z\right)\right)\right)=0 \end{array}$$

and then ∇ϕ(z)=s(z) proving Theorem 2.

## Appendix B. i-sgd Method Proofs and Details

### Appendix B.1. Proof of Corollary 1

The requirement C(θ)⪰0∀θ, ensures that the injected noise covariance is valid. The composite noise matrix is equal to ∑(θ)=Λ. Since $\nabla^{⊤}\sum \left(\theta \right)=\nabla^{⊤}\Lambda =0$ and $\eta P\left(\theta \right)=\Lambda^{-1}$ by construction, then Theorem 1 is satisfied.

### Appendix B.2. Proof of Optimality of Λ

Our design choice is to select $\lambda^{\left(p\right)}=\beta^{\left(p\right)}$. By the assumptions, the matrix B(θ) is diagonal, and consequently C(θ)=Λ−B(θ) is diagonal as well. The preconditioner Λ must be chosen to satisfy the positive semidefinite constraint, i.e., $C{\left(\theta \right)}_{ii}\geq 0\;\forall i,\forall \theta$. Equivalently, we must satisfy $\lambda^{\left(p\right)}-b_{j}\left(\theta \right)\geq 0\;\forall j\in I_{p},\forall p,\forall \theta$, where $I_{p}$ is the set of indices of parameters belonging to $p_{th}$ layer. By assumption 3, i.e., $\beta^{\left(p\right)}=\sum_{k\in I_{p}}b_{k}\left(\theta \right)$, it is easy to show that $b_{j}\left(\theta \right),j\in I_{p}$, is upper bounded as $b_{j}\left(\theta \right)\leq \beta^{\left(p\right)}$. To satisfy the positive semidefinite requirement in all cases the minimum valid set of $\lambda^{\left(p\right)}$ is then determined as $\lambda^{\left(p\right)}=\beta^{\left(p\right)}$.

### Appendix B.3. Algorithmic Details

In this section, we provide further details about the practical implementation of the proposed scheme. At any (discrete) time instant a minibatch version of the gradient is computed that is distributed, according to the hypotheses of the main paper, as g(θ)∼N(∇f(θ),2b(θ). Since we assumed that the second-order moment is a good approximation of the variance, we can estimate b(θ) as $\frac{1}{2}\left(g\left(\theta \right)⊙g\left(\theta \right)\right)$. In practice, we found that the following running average estimation procedure to be the most robust

(A1) $$b\left(\theta \right)\leftarrow \mu b\left(\theta \right)+\left(1-\mu \right)\frac{1}{2}\left(g\left(\theta \right)⊙g\left(\theta \right)\right)$$

where μ∈(0,1]. In all experiments we considered μ=0.5

After a warmup period, the various $\lambda^{\left(p\right)}$, layer per layer, are estimated as $\lambda^{\left(p\right)}=\sum_{k\in I_{p}}b_{k}\left(\theta \right)$ and kept constant until the end. The estimation procedure continues during sampling phase, as the quantity $\lambda^{\left(p\right)}-b\left(\theta \right)$ is necessary at every step. As the learning rate is derived as $\frac{2}{\lambda^{\left(p\right)}}$, we found that the usage of second-order moments instead of variances, and in certain cases temperature scaling, kept the simulated trajectories more stable.

## Appendix C. Methodology

We hereafter present additional implementation details.

### Appendix C.1. Regression Tasks, with Simple Models

For this set of experiments we considered, the baseline is obtained by running the adam optimizer for 20,000 steps with learning rate 0.01 and default parameters. At test time we use 100 samples to estimate the predictive posterior distribution, using Equation (3), for the *sampling* methods (i-sgd,sgld,sghmc,vsgd), with a keep-every value equal to 1000. The i-sgd and vsgd sampling methods are started from the baseline. For i-sgd we selected temperature 0.01, while for sghmc and sgld we do performed experiments for temperatures 1 and 0.01. We modified the implementation of vsgd as the original implementation produced unstable learning rates (as noticed also in [9]). A simple and effective solution we implement that we kept throughout the experimental campaigns is to divide the learning rate by the number of parameters (thus performing variational inference on a tempered version of the posterior). For sgld the learning rate decay is the one suggested in [2], with initial and finial learning rate equal to $10^{-6}$ and $10^{-8}$ respectively. For mcd we collected 1000 samples with standard dropout rate of 0.5. All our experiments use 10-splits. The considered batch size is 64 for all methods.

### Appendix C.2. Classification Task, ConvNet

For the LeNet-5 on mnist experiment, we do consider also the swag algorithm. At test time we use 30 samples for all methods. Baselines are again trained using adam optimizer for 20,000 steps with learning rate 0.01 and default parameters. For i-sgd and sghmc we collected samples for the different temperatures of 1 and 0.01. sgld has initial and final learning rates of $10^{-3}$ and $10^{-5}$. For all the sampling methods we do collect 100 samples with a keep-every of 10,000 steps. swag results are obtained by collecting the statistics over 300 epochs using adam optimizer and decreasing the learning rate every epoch in accordance with the original paper schedule [9]. drop results are obtained by training the networks with sgd, with learning rate 0.005 and momentum 0.5. The number of collected samples for this method is 1000. The batch size for all the methods is 128.

As explained in the main text, we performed an ablation study on the considered baselines. In Table A1 we do report the results for the additional variants obtained by early stopping (10,000 iterations instead of 20,000) baseline S, to ablate overfitting, and baseline L, by training for 30,000 iterations. Finally, we include the best performing baseline R, obtained starting from baseline, reducing the learning rate by a factor of 10 and training for 10,000 more iterations.

**Table A1:** Baselines comparison for classification on mnist dataset.

Method	acc	mnll	Mean $H_{0}$	ece	Mean $H_{1}$	Failed
baseline	9886.6667 ± 11.0252	352.6640 ± 20.8622	0.0353 ± 0.0058	0.0468 ± 0.0001	0.0019 ± 0.0003	0.0000
baseline l	9871.6667 ± 20.7579	389.7142 ± 79.0354	0.0378 ± 0.0051	0.0468 ± 0.0008	0.0025 ± 0.0006	0.0000
baseline s	9893.0000 ± 4.8990	339.8170 ± 7.9855	0.0392 ± 0.0042	0.0477 ± 0.0008	0.0024 ± 0.0001	0.0000
baseline r	9919.0000 ± 9.4163	242.7644 ± 17.0736	0.0303 ± 0.0001	0.0482 ± 0.0006	0.0021 ± 0.0002	0.0000

### Appendix C.3. Classification Task, Deeper Models

We here report details for the ResNet-18 on cifar10 experiments. The baseline is obtained with adam optimizer with learning rate 0.01 decreased by a factor of 10 every 50 epochs for a total of 200 epochs and weight decay of 0.05. For this set of experiments no temperature scaling was required. We could not find good hyperparameters for the sgld scheme. Concerning i-sgd, sghmc and vsgd the keep-every value is chosen as 10,000 and the number of collected samples is 30. For swag we used the default parameters described in [9]. Notice that for swag we performed the following ablation study: we trained the networks considering as loss function the joint log-likelihood and included or not the suggested weight decay of the original work [9]. From a purely Bayesian perspective no weight decay should be considered to be the information is implicit in the prior; however, we found that without the extra decay swag was not able to obtain competitive results. As underlined in Section 4.1, not necessarily a better posterior approximation translates into better empirical results.

### Appendix C.4. Definition of the Metrics

For regression datasets, we consider rmse and mnll. Consider a single datapoint $U_{i}=\left(x_{i},y_{i}\right)$, with $x_{i}$ the input of the model and $y_{i}$ the true corresponding output. The output of the model, for a single sample of parameters $\theta_{j}$, is ${\hat{y}}_{\theta_{j}}\left(x_{i}\right)$. rmse is defined as $\frac{1}{N}\sum_{i=1}^{N}\left|\right|y_{i}-\mu \left(x_{i}\right){\left|\right|}^{2}$, where $\mu (x_{i})$ is the empirical mean $\frac{1}{N_{MC}}\sum_{j=1}^{N_{MC}}{\hat{y}}_{\theta_{j}}\left(x_{i}\right)$. mnll is defined instead as $(\frac{1}{N}\sum_{i=1}^{N}\left(\frac{1}{2}\log\left(2\pi \sigma_{i}^{2}\right)+\frac{1}{2}\frac{\left|\right|y_{i}-\mu \left(x_{i}\right){\left|\right|}^{2}}{\sigma_{i}^{2}}\right)$, where $\sigma_{i}^{2}$ is the empirical variance.

For classification datasets, we consider acc,mnll and entropy. Consider a single datapoint $U_{i}=\left(x_{i},y_{i}\right)$, with $x_{i}$ the input of the model and $y_{i}$ the true corresponding label. The output of the model, for a single sample of parameters $\theta_{j}$, is the $N_{cl}$ vector $p_{\theta_{j}}\left(x_{i}\right)$. The averaged probability vector for a single sample is $p\left(x_{i}\right)=\frac{1}{N_{MC}}\sum_{i=1}^{N_{MC}}p_{\theta_{j}}\left(x_{i}\right)$.acc is defined as $\frac{1}{N}\sum_{i=1}^{N}1\left(\arg\max p\left(x_{i}\right)=y_{i}\right)$. mnll is computed as $\frac{1}{N}\sum_{i=1}^{N}\log\left(p_{y_{i}}\left(x_{i}\right)\right)$. Entropy, as stated in the main text, is instead computed according to $\frac{1}{N}\sum_{i=1}^{N}\left(\sum_{k=1}^{N_{cl}}p_{k}\left(x_{i}\right)\log\left(p_{k}\left(x_{i}\right)\right)\right)$.
